# Targeting the complement system in ANCA-associated vasculitis management

**DOI:** 10.1093/rheumatology/keaf539

**Published:** 2025-10-24

**Authors:** Kirsten de Groot

**Affiliations:** Med. Klinik III—Innere Medizin, Nephrologie, Rheumatologie, Sana Klinikum Offenbach GmbH, Offenbach, Germany; KfH Kidney Center, Offenbach, Germany

**Keywords:** ANCA, vasculitis, Wegener’s granulomatosis, autoinflammatory conditions, microscopic polyangiitis

## Abstract

ANCA-associated vasculitis (AAV) is a group of chronic multisystem inflammatory disorders affecting multiple organs throughout the body. Despite advances in AAV therapies, patients with AAV continue to experience higher rates of mortality than the general population. AAV outcomes can be improved by providing patients with rapid access to multidisciplinary medical teams and early treatment with glucocorticoid- (GC-) based therapy. However, GCs are associated with a high risk of toxicity. The phase 3 ADVOCATE trial demonstrates that blocking the alternative complement pathways using avacopan alongside immunosuppression enables patients with AAV to reduce their GC exposure without compromising efficacy or safety outcomes. ADVOCATE subgroup analyses show benefits for avacopan in patients with a wide range of AAV-related manifestations, disease stages and ages, with the greatest benefits in patients with kidney impairment, lung manifestations and AAV relapse.

## Graphical Abstract

**Graphical abstract:**
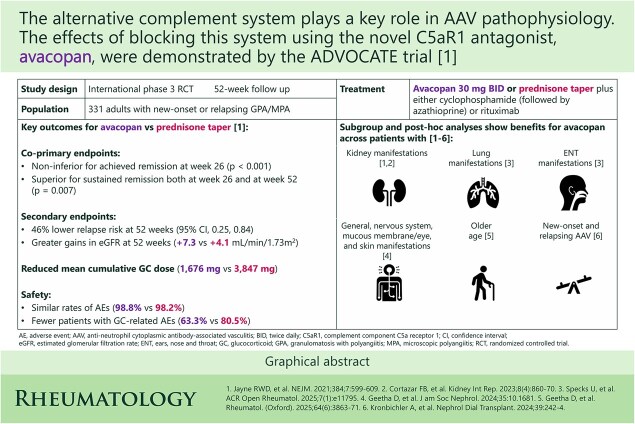

Rheumatology key messages

- The alternative complement system plays a key role in AAV pathophysiology.
- Avacopan (a complement C5a receptor 1 antagonist) enables AAV patients to reduce their glucocorticoid exposure.
- Post-hoc analyses of ADVOCATE trial data show benefits for avacopan across various patient subgroups.

## Introduction

ANCA-associated vasculitis (AAV) is a group of chronic inflammatory disorders comprising granulomatosis with polyangiitis (GPA), microscopic polyangiitis (MPA) and eosinophilic GPA (EGPA) [1, 2]. Advances in therapies over recent decades have extended survival times for AAV patients from an average of 5 months in 1958 [3] to a median 17.8 years (95% CI 15.7, 20 years) in 2023 [4]. Despite this, patients continue to experience limited survival compared with the general population, with excess cumulative mortality ranging from 14% at 1 year to 20% at 10 years and 29% at 15 years [4]. Negative prognostic factors for death among people with AAV include advanced age (>50–55 years), male sex, lower platelet levels and lower estimated glomerular filtration rates (eGFRs).

National health datasets for 1420 vasculitis patients across the UK and Ireland show that AAV mortality rates can be reduced by ensuring patients have timely access to integrated care and vasculitis expertise [5]. The hazard ratio (HR) for mortality was 0.59 (95% CI 0.37, 0.93) among patients seen by vasculitis experts within 1 week of diagnosis and 0.72 (95% CI 0.50, 1.10) for patients with access to a joint or parallel multidisciplinary team of doctors and nurse specialists. Results from this study support AAV treatment guidelines, which recommend managing AAV patients using a multidisciplinary team of healthcare professionals at centres with, or with access to, vasculitis expertise [6, 7].

Recommended treatment for MPA and GPA includes induction therapy to rapidly control active disease followed by less aggressive maintenance strategies to reduce the risk of AAV relapse [6, 7]. This typically includes a combination of cytotoxic drugs (e.g. rituximab [RTX] or cyclosphosphamide [CYC]) alongside glucocorticoids (GCs) and/or avacopan [6, 7]. Numerous randomized clinical trials (RCTs) have investigated the optimal use of cytotoxic drugs in patients receiving induction and/or maintenance therapy for AAV [8–19], whereas only two RCTs (PEXIVAS [20] and LoVAS [21]) have evaluated a reduced dosing scheme for GC. The ADVOCATE trial is the first phase 3 RCT to investigate the safety and efficacy of avacopan (a novel complement 5a receptor 1 [C5aR1] antagonist) as adjunctive therapy to RTX or CYC in patients with GPA/MPA and reduced or no GC exposure [22].

## Role of the complement system in AAV pathophysiology

Kidney manifestations, typically characterized by pauci-immune glomerulonephritis (GN), are a common feature of AAV [23]. Kidney biopsies taken from AAV patients with GN showed complement component 3 (C3) deposits in the glomerular tuft, peritubular capillaries and/or venules in 39 of the 43 tissue samples (90.7%), with the majority of deposits in the glomerular tuft and peri tubular capillaries [23]. Furthermore, blood samples from 66 patients with active AAV and 54 patients with AAV in remission found that plasma levels of C3a, C5a, soluble C5b-9 and Bb were higher when AAV was active [24]. This suggests a role for the complement system in AAV pathophysiology.

The complement system can be activated via three main pathways: the classical pathway, the lectin pathway and the alternative pathway [25–29]. The classical pathway is activated when antigen bound to immunoglobulin (Ig) M or G, binds to complement protein C1q, whereas the lectin pathway is activated when lectins, collectins or ficolins bind to carbohydrates on the surface of pathogens. Unlike the other two pathways, the alternative pathway is in a constant state of low-level activation, which enables rapid complement expansion in response to pathogens. If unregulated, all three pathways lead to the generation of C3b, the activated component of C3. The binding of C3b to components of the C3 convertases results in the formation of the C5 convertases, which cleave C5 into C5a and C5b, ultimately leading to the formation of the inflammatory protein, C5a, and the membrane attack complex (MAC), C5b-9.

AAV is caused by the production of antibodies directed against one of two neutrophil enzymes—myeloperoxidase (MPO) and proteinase 3 (PR3) [23, 24, 29]. Patients with GPA usually have ANCA against PR3, whereas more than half of the patients with MPA have ANCA against MPO [1, 30, 31]. Preclinical studies in mice with MPO-induced GN found that blocking the alternative pathway by C5 knockout prevented the development of GN, whereas blocking the classical and lectin pathway via C4-knockout had little or no effect [32]. Similarly, blocking the alternative complement pathway using a C5-inhibiting monoclonal antibody protected against the development of GN and reduced the formation of glomerular crescent by >80% 1 day after disease induction [33].

**Figure 1.**
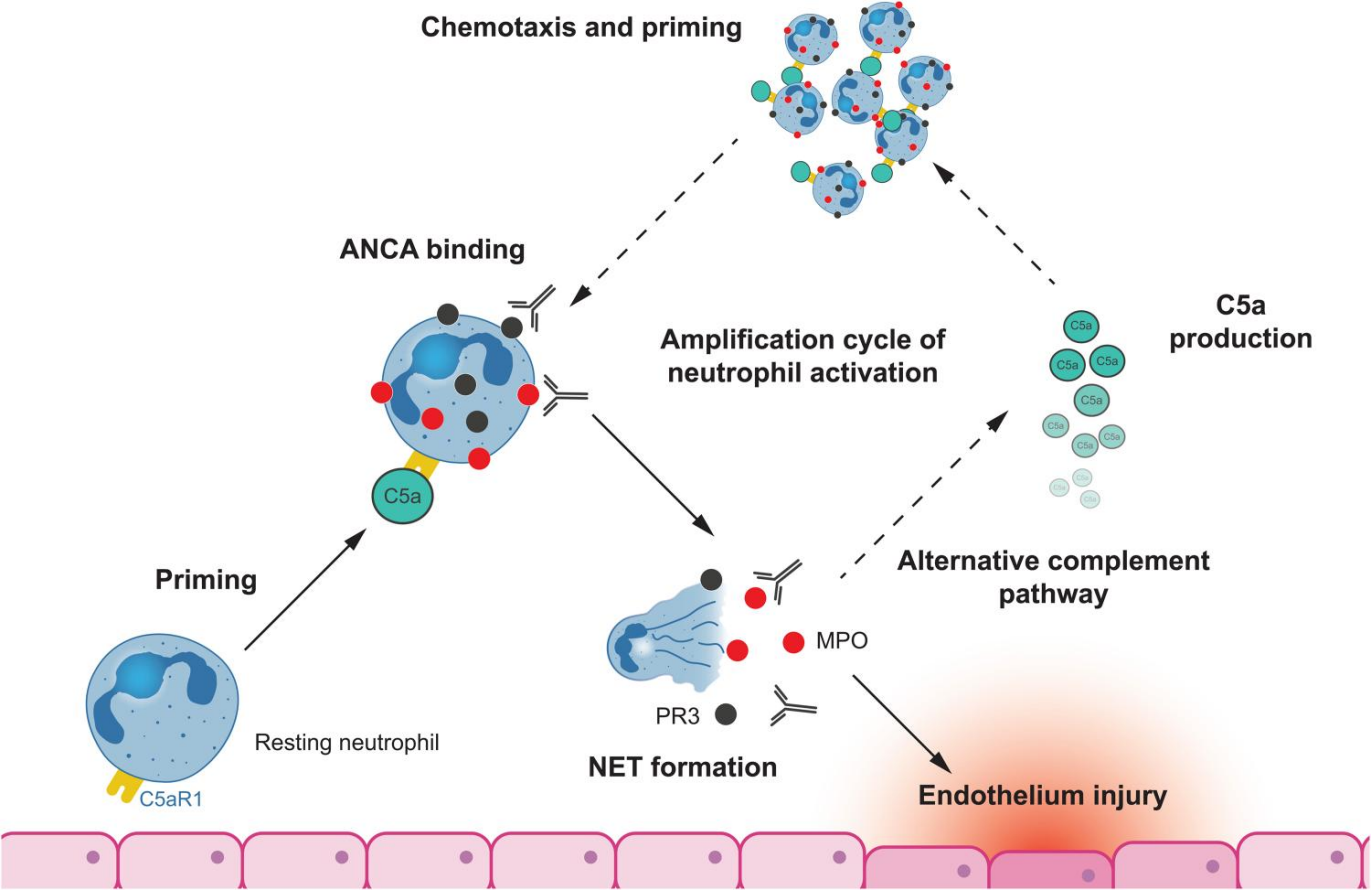
Role of the alternative complement system in the pathophysiology of AAV. Neutrophils primed by inflammatory stimuli express MPO or PR3, which bind to ANCA triggering NET formation and activation the alternative complement system leading to C5a production. C5a causes chemotaxis of immune cells (including neutrophils) and binds to C5aR1 on neutrophils, further priming them for activation by ANCA in an amplification loop that results in sustained inflammation. AAV: ANCA-associated vasculitis; ANCA: anti-neutrophil cytoplasmic antibody; C5a: complement component 5a; C5aR1: complement component 5a receptor 1; MPO: myeloperoxidase; NET: neutrophil extracellular trap; PR3: proteinase 3

Subsequent studies found that C5a and its receptor, C5aR1, on neutrophils play a key role in priming neutrophils for activation by ANCAs [34]. The binding of ANCA to MPO or PR3 on the surface of primed neutrophils triggers neutrophil extracellular trap (NET) formation which activates the alternative complement system leading to the production of C5a (Fig. 1). C5a causes chemotaxis of immune cells (including neutrophils) and binds to C5aR1 on neutrophils, priming them for activation by ANCA in a cycle which rapidly amplifies neutrophil activation resulting in sustained inflammation.

## Avacopan

Avacopan is a novel, small molecule human C5aR1/CD88 antagonist approved for use in adults with severe, active GPA or MPA in combination with standard therapy including GC according to the FDA [35] or a RTX- or CYC-based regimen according to the EU [36]. A pivotal pre-clinical study found that avacopan blocked human C5aR in mice with MPO antibody-induced GN, significantly reducing the number of glomerular crescents from approximately 30.4% to 3.3% and reducing levels of hematuria (*P* < 0.001), proteinuria (*P* < 0.01) and leukocyturia (*P* < 0.001) [37]. Avacopan was subsequently tested in a phase 1 dose-finding study [26], two phase 2 clinical trials (CLEAR [38] and CLASSIC [39]) and the phase 3 ADVOCATE trial [22].

### Efficacy and safety of avacopan in patients with MPA/GPA

ADVOCATE was a 52-week double-blind, double-dummy, active-controlled, international phase 3 trial conducted in 331 adults with newly diagnosed or relapsing GPA or MPA [22]. Patients were randomized to receive either oral avacopan 30 mg BID (*n* = 166) or oral prednisone 60 mg/day tapered to discontinuation over 20 weeks (*n* = 165). All patients received background induction immunosuppressive treatment including either RTX weekly for 4 weeks or CYC followed by azathioprine at the discretion of the study investigator. Patients with worsening AAV or reasons unrelated to AAV could also be treated with non-study supplied GCs.

**Figure 2.**
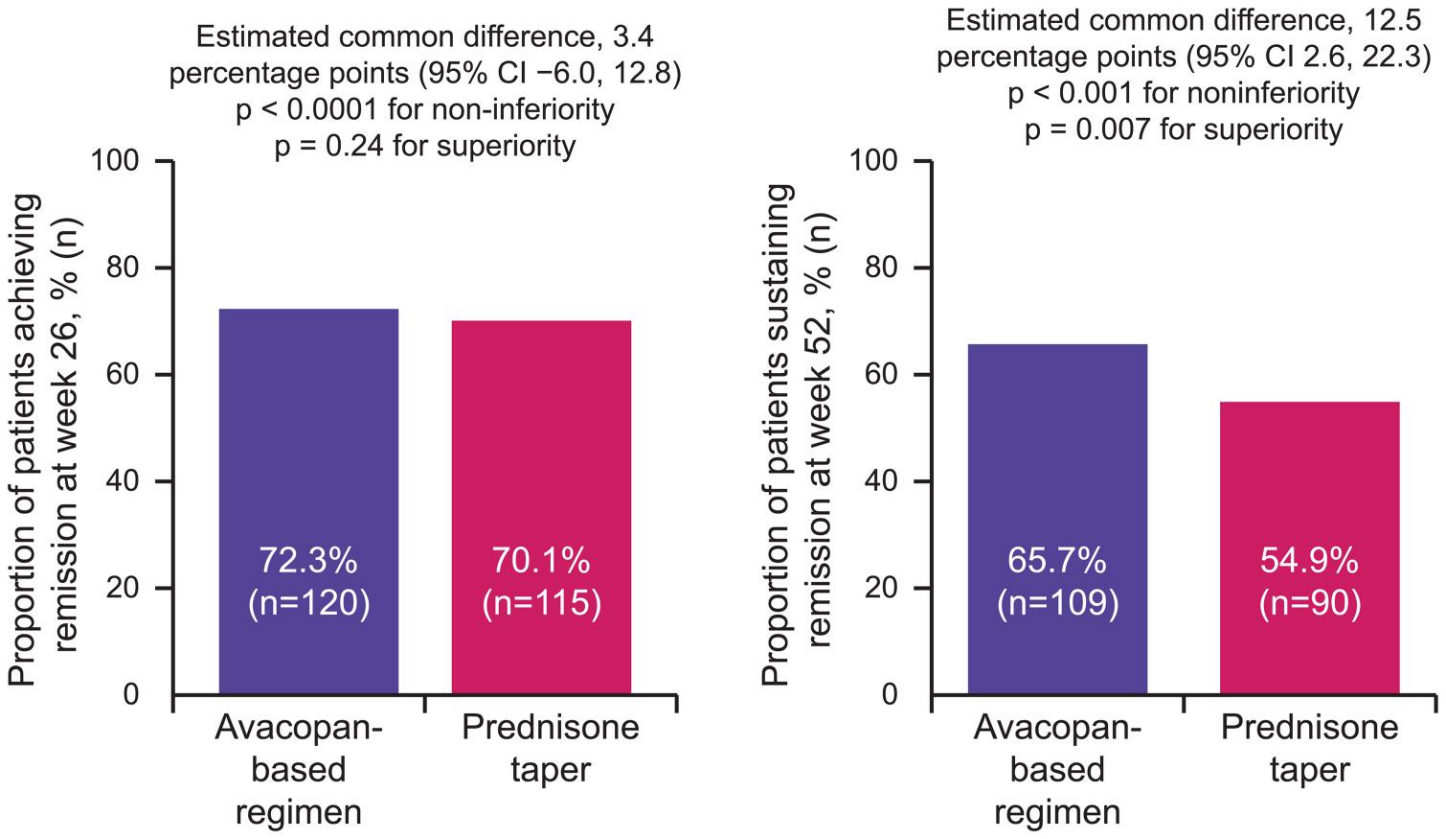
Proportion of patients receiving avacopan *vs* prednisone taper who achieved AAV remission at week 26 and sustained remission at week 52 (primary endpoints) in the ADVOCATE trial [22]. 166 patients in the avacopan group and 164 patients in the prednisone taper group were included in endpoint analyses at weeks 26 and 52. Remission at week 26 was defined as BVAS = 0 and no GC for 4 weeks before week 26. Sustained remission at week 52 was defined as remission both at week 26 and at week 52 (BVAS = 0 and no receipt of GC for 4 weeks before week 52). AAV: anti-neutrophil cytoplasmic antibody-associated vasculitis; GC: glucocorticoid; n: number of patients

The first primary endpoint for ADVOCATE (achieving clinical remission at week 26, defined as BVAS = 0 and no receipt of GCs for 4 weeks before week 26) was achieved by 72.3% of patients in the avacopan group compared with 70.1% of the prednisone taper group (estimated common difference 3.4 percentage points [95% CI −6.0, 12.8]; *P* < 0.001 for noninferiority; *P* = 0.24 for superiority) (Fig. 2). The second primary endpoint (sustained remission at week 52, defined as remission [BVAS = 0] at week 26 and at week 52 and no receipt of GCs for 4 weeks before week 52) was achieved by 65.7% of the avacopan group compared with 54.9% of the prednisone taper group (estimated common difference 12.5 percentage points [95% CI 2.6, 22.3]; *P* < 0.001 for noninferiority; *P* = 0.007 for superiority) (Fig. 2).

**Figure 3.**
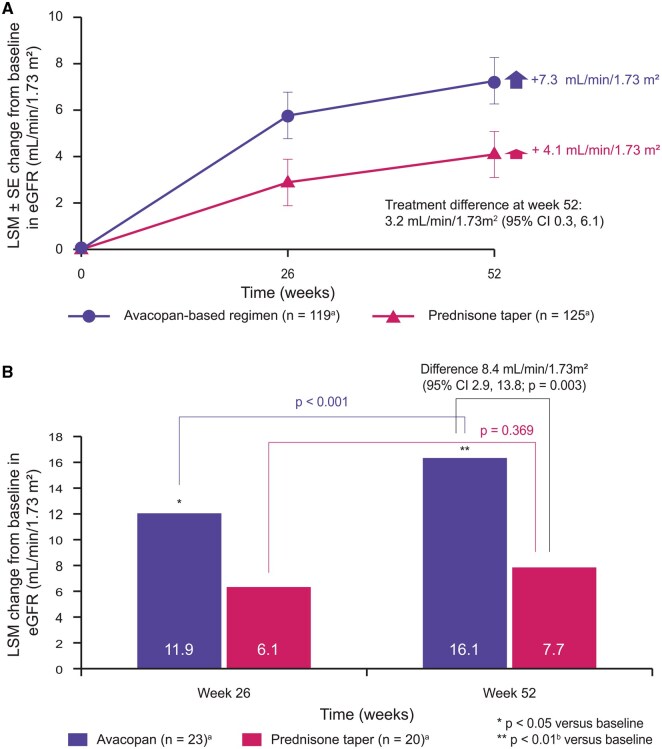
Changes in eGFR in all patients receiving avacopan *vs* prednisone taper in the ADVOCATE trial [22] (**A**) and in the subgroup of patients with baseline eGFR ≤20 ml/min/1.73 m^2^ [40] (**B**). ^a^Patient numbers shown are for week 52 only. In the ADVOCATE trial (**A**) patient numbers in avacopan and prednisone taper groups were 131 and 134, respectively at baseline, and 121 and 127, respectively, at week 26. In the subgroup analysis (**B**), patient numbers in avacopan and prednisone taper groups were 27 and 23, respectively at baseline, and 24 and 19, respectively, at week 26. ^b^Measured by mixed effects models for repeated measures with treatment group, study visit, and treatment-by-visit interaction as factors and baseline as covariate. There was no pre-specified plan for adjustment of CIs for multiplicity of the secondary endpoints; point estimates and 95% CI only are presented, and no definite conclusions can be drawn from these data. eGFR: estimated glomerular filtration rate; GC: glucocorticoid; LSM: least squares mean; SEM: standard error of mean

Secondary endpoints in the ADVOCATE trial showed that the absolute risk of AAV relapse over 52 weeks was 46% lower with avacopan (10.1%) *vs* prednisone (21.0%) (HR 0.46 [95% CI 0.25, 0.84]), despite considerably reduced GC exposure in the avacopan group (mean cumulative GC dose 1676 mg *vs* 3847 mg). Furthermore, avacopan treatment enabled greater gains in eGFR at week 52 than prednisone taper (treatment difference +3.2 ml/min/1.73 m^2^ [95% CI 0.3, 6.1]) (Fig. 3A) [22]. Although the difference between treatment groups was considerable, there was no pre-specified plan to adjust CIs for multiplicity and so statistical significance of the secondary endpoints could not be determined.

The overall safety profile for avacopan appeared favorable in patients with GPA/MPA, with a similar subject incidence of adverse events (AEs) in both treatment groups at 52 weeks (98.8% with avacopan; 98.2% with prednisone taper) [22]. The frequencies of serious AEs (SAEs), life-threatening AEs and death were numerically lower in the avacopan group (42.2%, 4.8% and 1.2%, respectively) compared with the prednisone taper group (45.1%, 8.5%, and 2.4%, respectively). Importantly, the incidence of AEs possibly related to GCs was 66.3% in the avacopan group compared with 80.5% in the prednisone group (difference, −14.2 percentage points [95% CI −23.7, −3.8]).

A subsequent analysis of pooled safety data from the ADVOCATE [22], CLEAR [38] and CLASSIC trials [39] found that, after adjusting for treatment exposure, rates per 100 patient years of first incidence AEs (1099.8 *vs* 1251.7; difference −151.9 [95% CI −218.6, −85.3]), infection-related AEs (142.2 *vs* 166.6; difference −24.3 [95% CI −48.5, −0.1]), neutropenia/lymphopenia (22.6 *vs* 34.2; difference −11.6 [95% CI −22.0, −1.2]), and SAEs (70.7 *vs* 91.5; difference −20.8 [95% CI −38.3, −3.3]) per 100 patient years were lower in patients receiving avacopan with a reduced-dose GC regimen (*n* = 239) compared with those receiving standard (non-avacopan) treatment (*n* = 200). These benefits were likely due to reduced GC exposure in the avacopan group *vs* non-avacopan group. Rates of AEs related to liver function (18.4 *vs* 17.4; difference +1.0 [95% CI −7.2, 9.2]) and hypersensitivity reactions (68.8 *vs* 61.8; difference +6.9 [95% CI −8.7, 22.6]) were slightly higher in the avacopan *vs* the non-avacopan group, while rates of AEs leading to study drug discontinuation (21.7 *vs* 21.5; difference +0.2 [95% CI −8.8, 9.2]) were similar between groups.

### Efficacy and safety of avacopan in different patient populations

#### Kidney manifestations

The ADVOCATE trial showed greater gains in eGFR at week 52 in the avacopan group compared with prednisone taper (Fig. 3A) [22], with more pronounced gains in the subgroup of patients with eGFR ≤20 ml/min/1.73 m^2^ at baseline (*n* = 50) (treatment difference +8.4 ml/min/1.73 m^2^ [95% CI 2.9, 13.8]; *P* = 0.003) (Fig. 3B) [40]. The continued least squares mean (LSM) increase in eGFR between weeks 26 and 52 in the avacopan group (*P* < 0.001) but not the prednisone taper group (*P* = 0.369) suggests avacopan was associated with sustained kidney repair and reduced inflammation throughout the trial. This suggests avacopan may be particularly useful in patients with impaired kidney function.

#### Lung manifestations

Of the 331 patients in the ADVOCATE trial, 142 had lung manifestations at baseline (42.9%), most commonly nodules or cavities (52.1% of patients in the avacopan group *vs* 49.3% in the prednisone taper group), infiltrates (43.7% *vs* 52.1%) and wheezing (15.5% *vs* 14.1%) [41]. Lung manifestations rapidly decreased during the first 4 weeks of treatment in both groups, persisting in 0% of patients in the avacopan group and 4.4% of patients in the prednisone taper group at week 52. AAV remission at week 26 was achieved by a numerically higher proportion of patients with lung involvement receiving avacopan (73.2%) *vs* prednisone taper (66.2%), with 67.6% and 53.5%, respectively, sustaining remission at week 52 (difference 14.1 [95% CI −1.8, 30.0]). Among patients with lung manifestations, the mean dose of GC from any source was consistently lower at all timepoints in the avacopan group, with cumulative doses of 2219 mg in the avacopan group and 3926 mg in the prednisone taper group during the 52-week trial. Relapse rates over 52 weeks were 8.7% in the avacopan group *vs* 23.5% with prednisone taper, while rates of lung relapse were 4.3% and 10.3%, respectively.

Of the 12 patients with diffuse alveolar haemorrhage (DAH) at baseline, 11 had GPA, 10 received RTX background therapy, 8 were male, 8 had PR3-ANCA and 6 were newly diagnosed. Overall, 4 of the 5 avacopan-treated patients and 5 of the 7 patients receiving prednisone taper achieved remission at week 4 (BVAS 0), all of which sustained remission at week 26 (BVAS 0 and no use of GCs for the treatment of GPA or MPA within 4 weeks prior to and including the week 26 visit). Mean cumulative GC dose was considerably lower in the avacopan group, both at baseline (week −2–0; 300 mg *vs* 1945 mg) and during weeks 0–4 (625 mg *vs* 1627 mg). This suggests avacopan might be particularly useful for the treatment of DAH, which is associated with progression to respiratory failure.

#### ENT manifestations

In the ADVOCATE trial, 144 patients (44%) had ENT manifestations at baseline. Of these, 2.9% of the avacopan group and 7.6% of the prednisone taper group had active ENT manifestations at week 52, with the proportions of affected patients decreasing at a similar rate over time in each group [41]. Remission was achieved by 72.0% of the avacopan group *vs* 71.0% of the prednisone group at week 26 and in 62.7% *vs* 53.6% of patients, respectively, at week 52.

#### General, nervous system, mucous membrane/eye and skin manifestations

Among the 331 patients in the ADVOCATE trial, 225 (68.2%) had active general manifestations consistent with a chronic inflammatory disorder at baseline (including myalgia, arthralgia/arthritis, fever ≥38°C and weight loss ≥2 kg), 69 (20.9%) had nervous system manifestations (e.g. headache, meningitis, cerebrovascular accident, organic confusion, spinal cord lesion, cranial nerve palsy, sensory peripheral neuropathy, mononeuritis multiplex, etc.), 66 (20.0%) had mucous membrane/skin manifestations (mouth ulcers, significant proptosis, scleritis/episcleritis, conjunctivitis/blepharitis/keratitis, blurred vision, sudden visual loss, uveitis, etc.), and 47 (14.2%) had skin involvement (infarct, purpura, ulcer, other skin vasculitis, etc.) [42]. Substantial improvements in the control of active disease in each of these organ systems were achieved in both treatment groups, with the avacopan group achieving numerically higher remission rates at week 52 for general (97.3% *vs* 96.5%), nervous system (100% *vs* 93.5%), and mucous membrane/eye manifestations (100% *vs* 95.0%), but not skin manifestations (83.3% *vs* 100%).

#### Patients aged ≥65 years

**Figure 4.**
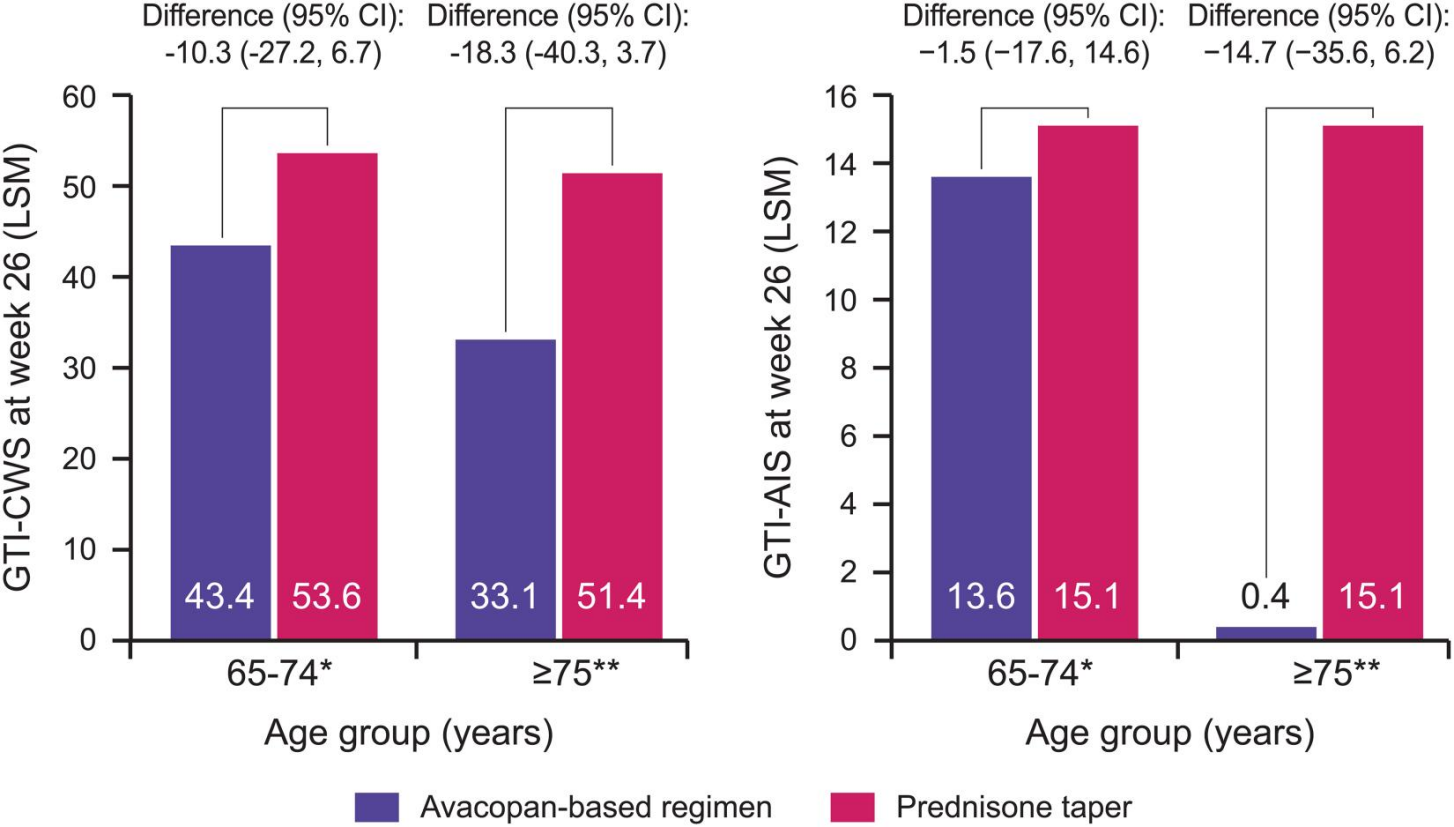
Differences in GC toxicity scores at week 26 between patients aged 65–74 years and ≥75 years receiving avacopan *vs* prednisone taper in the ADVOCATE trial [43]. Patient numbers for the avacopan and prednisone taper groups were *n* = 60 and *n* = 49, respectively, for patients aged 65–74 years, and *n* = 26 and *n* = 25, respectively, for patients aged ≥75 years. There was no pre-specified plan for adjustment of CIs for multiplicity of the secondary endpoints; point estimates and 95% CIs only are presented, and no definite conclusions can be drawn from these data. *Median all-source GC dose: avacopan-based regimen 575 mg *vs* GC-based regimen 3055 mg; **Median all-source GC dose: avacopan-based regimen 588 mg *vs* GC-based regimen 2840 mg. AIS: aggregate improvement score; CWS: cumulative worsening score; GC: glucocorticoid; GTI: glucocorticoid toxicity index; LSM: least squares mean

A post-hoc analysis of ADVOCATE trial data found that, compared with the prednisone taper group, the avacopan group had a 5.3-fold reduction in median all-source GC dose in the subgroup of patients aged 65–74 (*n* = 109) and a 4.8-fold reduction in the subgroup aged ≥75 years (*n* = 51) [43]. GC toxicity index (GTI) as assessed by the cumulative worsening score (CWS) and aggregate improvement score (AIS) at week 26 was numerically lower in the avacopan *vs* prednisone taper groups across both patient age groups, with the largest difference occurring in patients aged ≥75 years (GTI-CWS LSM difference −18.3 [95% CI −40.3, 3.7] and AIS LSM difference −14.7 [95% CI −35, 6.2]) (Fig. 4). The proportions of AEs and SAEs were broadly similar between treatment groups in both age groups. However, the proportion of SAEs related to infections was considerably lower in patients aged ≥75 years receiving avacopan (15.4%) *vs* prednisone (24.0%).

#### New-onset *vs* relapsing AAV

The ADVOCATE trial included 229 patients with new-onset AAV and 101 with relapsing AAV at baseline [44]. A similar proportion of patients with newly diagnosed AAV achieved remission at week 26 in the avacopan group (66.1%) and the prednisone taper group (66.7%) (difference −0.6% [95% CI −12.8, 11.7]), whereas results among patients with relapsing disease were numerically favorable for avacopan (86.3% *vs* 78.0%; difference 8.3% [95% CI −6.6, 23.1]). The proportion of patients achieving sustained remission at week 52 was similar for both treatment groups among patients with new-onset disease (60.9% *vs* 57.9%; difference [95% CI]: 3.0% [−9.7, 15.7]), whereas avacopan was more effective among patients with relapsing disease (76.5% *vs* 48.0%; difference 28.5% [95% CI 10.4, 46.6]). Compared with prednisone taper, the proportion of patients who experienced relapse after remission at any time was lower with avacopan in patients with newly diagnosed (8.2% *vs* 18.2%) and relapsed (14.6% *vs* 27.7%) AAV at baseline. The overall, all-source median GC dose at 52 weeks was 625 mg in the avacopan group *vs* 3048 mg in the prednisone taper group for newly diagnosed AAV, and 500 mg *vs* 3139 mg for relapsed AAV.

## Summary and conclusions

Despite advances in AAV therapies, patients with AAV continue to experience higher rates of mortality than age- and gender-matched controls [4]. AAV outcomes can be improved by ensuring patients have rapid access to multidisciplinary medical teams [5] and early treatment with targeted therapies that enable reduced exposure to GC [22, 44, 45]. Early studies demonstrate a key role for the alternative complement system in the pathophysiology of AAV [23, 24, 27, 29, 33, 34, 37]. Consistent with this observation, results from the phase 3 ADVOCATE trial show that the adjunctive use of the C5aR1 antagonist, avacopan, alongside RTX or CYC enables patients with GPA/MPA to considerably reduce their GC exposure without compromising efficacy or safety outcomes [22, 40, 41, 43, 44]. Post-hoc analyses of ADVOCATE trial data demonstrate benefits for avacopan across diverse patient subgroups, including those with new-onset and relapsing AAV [44], elderly patients [43] and patients with manifestations related to the kidney [40], lung [41], ENT [41], chronic inflammation [42], nervous system [42], mucous membrane/eyes [42] and skin [42]. The benefits of avacopan were particularly pronounced in patients with kidney impairment (eGFR ≤20 ml/min/1.73 m^2^) [40], lung disease [41] and AAV relapse [44].

## Data Availability

No new data were generated or analysed in support of this research.
